# Supplementary material for: CX-5461 is a DNA G-quadruplex stabilizer with selective lethality in BRCA1/2 deficient tumours
Source: Nat Commun. 2017 Feb 17;8:14432. doi: 10.1038/ncomms14432 (PMC5321743; doi:10.1038/ncomms14432)
Supplement: Supplementary Information — Supplementary Figures and Supplementary Tables. [file ncomms14432-s1.pdf]

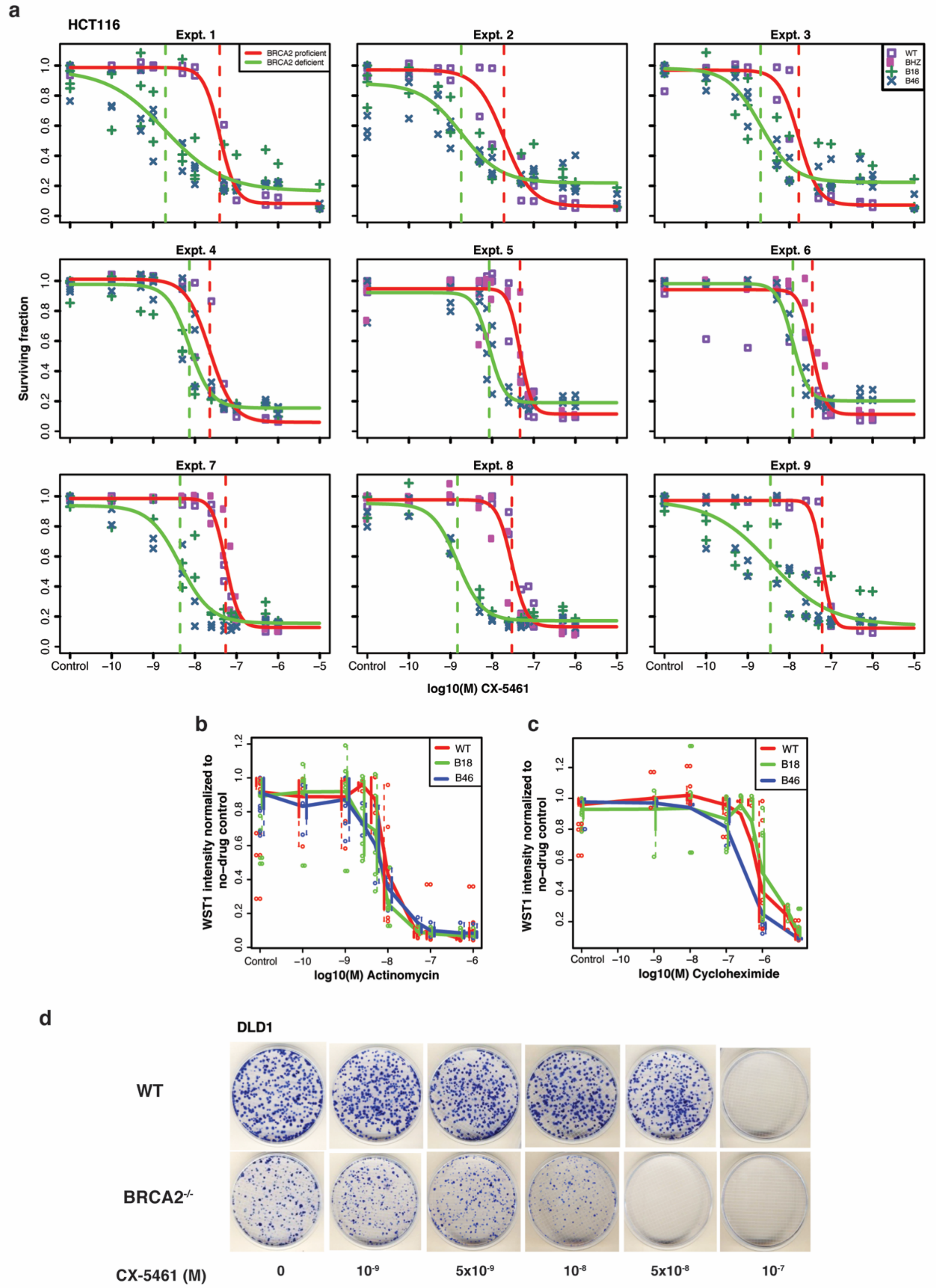

Supplementary Figure 1. (Relative to Figure 1) BRCA2 deficient cells are specifically sensitive to CX-5461.
(a) BRCA2 deficient HCT116 are more sensitive to CX-5461 than isogenic BRCA2 WT cells. Drug sensitivity to CX-5461 was compared between BRCA2 proficient and deficient cells in HCT116. Data from BRCA2 proficient cell lines (WT and BRCA2 heterozygous) were combined, and data from BRCA2 deficient (B18 and B46) were also combined. 9 independent WST-1 assay results were summarized (each with internal replicates, mean number of internal replicates=3).

The mean IC50 ratio across the 9 experiments was 8.997 (95%CI, 5.1-16.2). A t-test of the differences between BRCA2 proficient and deficient cell lines across 9 experiments yields $t(8)=8.76$, $p = 0.000023$. A simple non-parametric test (Wilcoxon sign test) for the differences in IC50 across the nine experiments yields $p = 0.0039$. Thus there is strong statistical evidence of a consistent difference in IC50 dose-response values for the BRCA2 deficient and proficient cell lines for the drug CX-5461.

(b and c) BRCA2 knockout cells are not more sensitive to actinomycin (b) and cycloheximide (c). The dose sensitivity in HCT116 is displayed by WST-1 assay, and the results of all 3 experiments are summarized in isogenic cell line pairs by the green (BRCA2 deficient) and red (BRCA2 wild type) super-smoother curve fit lines (See statistical methods). Drug treatment time is 6 days.

(d) BRCA2 knockout DLD1 cells are highly sensitive to CX-5461 shown by clonogenic assay. Drug treatment time is 12 days. Experiments were repeated twice and similar results were obtained.

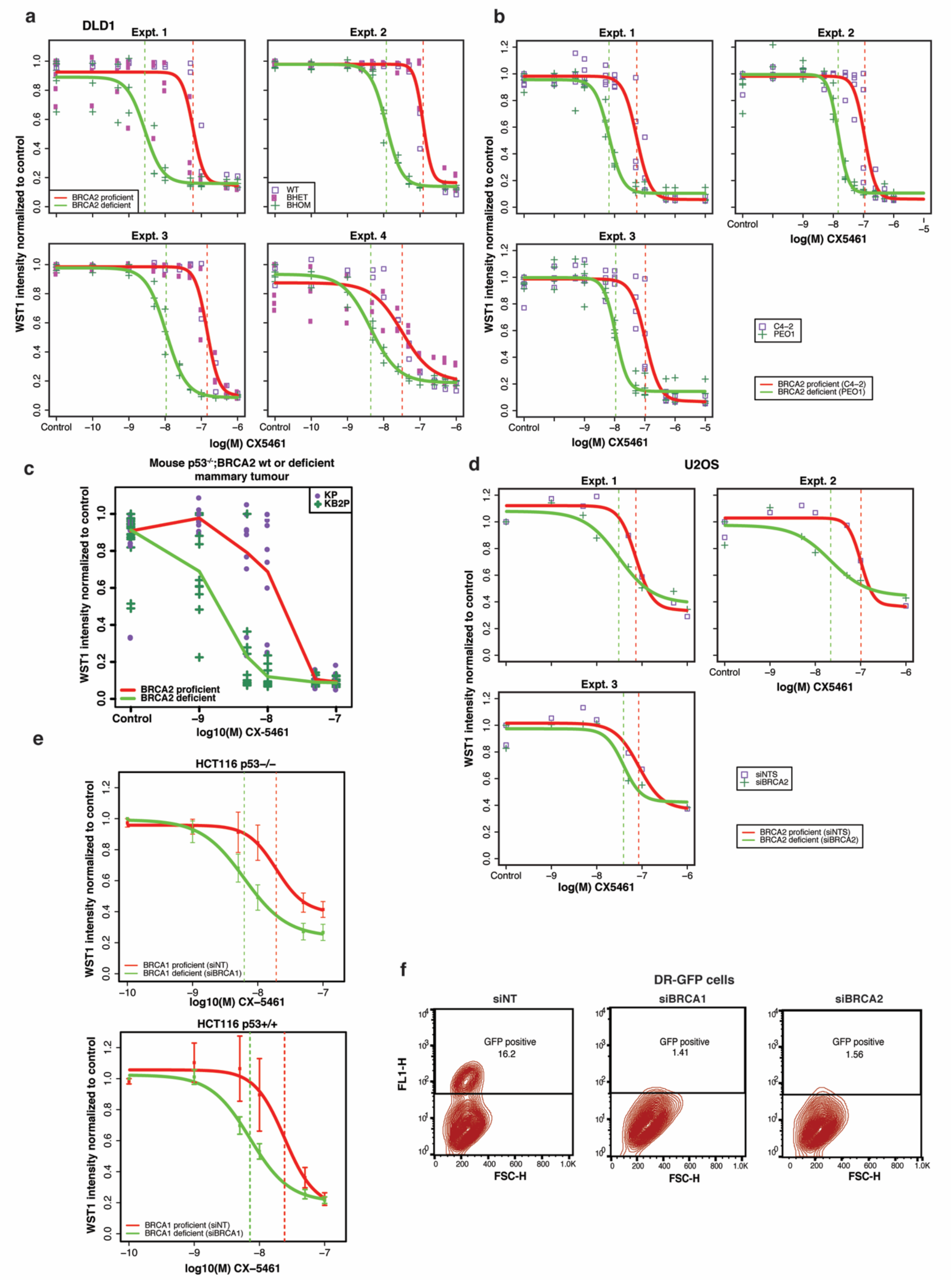

Supplementary Figure 2. (Relative to Figure 1) BRCA2 deficient cells are highly sensitive to CX-5461 in different cell lines.

(a) BRCA2 deficient DLD1 cells are more sensitive to CX-5461 than BRCA2 WT isogenic cells shown by WST-1 assay. 4 independent WST-1 assay results demonstrated the higher sensitivity of *BRCA2*$^{-/-}$ cells to CX-5461 relative to BRCA2 proficient cells (*BRCA2*$^{+/+}$ and *BRCA2*$^{+/-}$*)* in DLD1 (each with internal replicates, mean number of internal replicates=3). The

mean IC50 difference across the 4 experiments was 12.2 (95%CI, 6.03-25.1). A t-test of the 4 experiments, testing the hypothesis of no difference in IC50 values for BRCA2 proficient and deficient DLD1 cells yields $t(3)=11.14$, $p = 0.0015$. Thus we reject the null hypothesis and conclude that there is sufficient statistical evidence to suggest a difference in IC50 values for the BRCA2 proficient and deficient genotypes of the DLD1 cell line.

(b) BRCA2 deficient PEO1 ovarian cancer cells are more sensitive to CX-5461 than C4-2 revertant BRCA2 WT control cells. Three WST-1 assay results exhibited the higher sensitivity of *BRCA2*$^{-/-}$ (PEO1) cells to CX-5461 relative to BRCA2 proficient cells (C4-2). The mean IC50 difference across the 3 experiments was 8.46 (95%CI, 6.28-11.4) on the raw dose scale. A t-test of the 3 experiments, testing the hypothesis of no difference in IC50 values for BRCA2 proficient and deficient PEO1-derived cells yields $t(2)=30.8$, $p = 0.0011$. Thus we reject the null hypothesis and conclude that there is sufficient statistical evidence to suggest a difference in IC50 values for the BRCA2 proficient (C4-2) and deficient (PEO1) genotypes of the PEO1 cell line.

(c) BRCA2 knockout mouse breast tumor cells were highly sensitive to CX-5461 as demonstrated by WST-1 assay. The results of all 3 experiments are summarized in isogenic cell line pairs by the green (BRCA2 deficient) and red (BRCA2 wild type) super-smoother curve fit lines (See statistical methods).

(d) BRCA2 deficient (knocking down by siRNA) U2OS cells are more sensitive to CX-5461 than non-targeting control cells. BRCA2 was knocked down 3 times with siRNA in U2OS cells and drug sensitivity to CX-5461 was compared between cells with BRCA2 knocked down and non-targeting control. The mean IC50 difference across the 3 experiments was 2.89 (95%CI, 1.024-8.152) on the raw dose scale. A t-test of the 3 experiments, testing the hypothesis of no difference in IC50 values for BRCA2 proficient and deficient U2OS cells yields $t(2)=4.40$, $p = 0.048$. Thus we reject the null hypothesis and conclude that there is sufficient statistical evidence to suggest a difference in IC50 values for the BRCA2 proficient (siNTS) and deficient (siBRCA2) genotypes of the U2OS cell line.

(e) Increased drug sensitivity to CX-5461 was observed in BRCA1 knock down (with 4 different individual siRNA for BRCA1) *p53*$^{+/+}$ and *p53*$^{-/-}$ HCT116 cells by WST-1 assay (4 days in drug). p-value (by omnibus F-test) is 1.1 e-07 for *p53*$^{+/+}$ HCT116 and 1 .5e-08 for *p53*$^{-/-}$ HCT116 cells, comparing non-targeting control siRNA vs. all 4 siRNA for BRCA1. Efficiency of BRCA1 and BRCA2 knockdown was supported by western blotting and the decrease of HR rate (supplementary fig. 2F). Dashed lines indicate IC50.

(f) Knocking down BRCA1 and BRCA2 with siRNA significantly reduced HR rate comparing with siRNA for non-targeting control. DR-GFP U2OS cells were transfected with siRNA and ISce-I plasmid. After 48hrs, FACS analysis was performed to detect the percentage of GFP positive cells, which indicate HR rate.

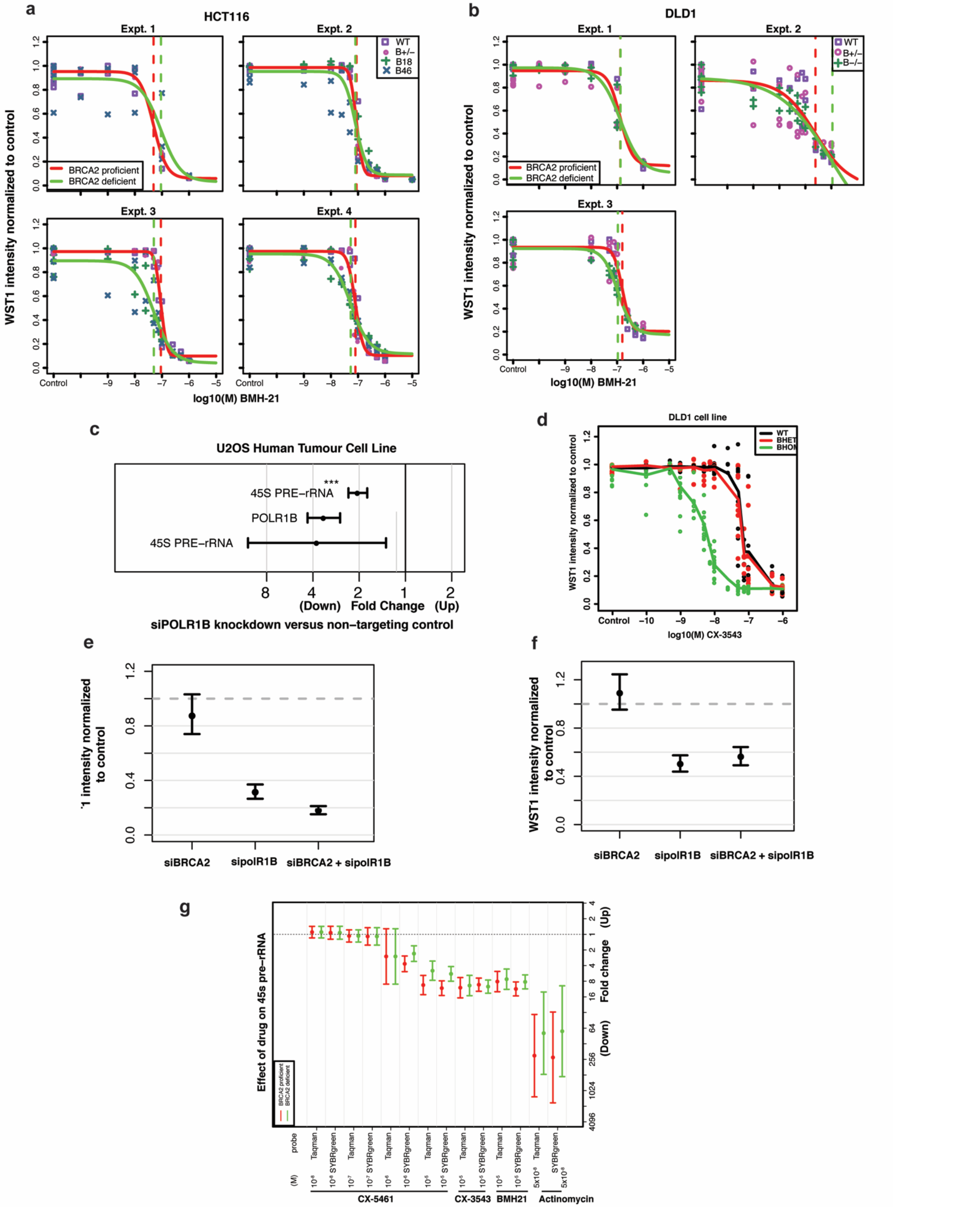

Supplementary Figure. 3. The selective toxicity of CX-5461 to BRCA2 deficiency is not due to rDNA transcription

inhibition.

(a) Drug sensitivity to BMH-21 was compared between BRCA2 proficient and deficient cells in HCT116. 4 independent WST-1 experiments were performed. The mean IC50 difference across the 4 experiments was 1.12 (95%CI, 0.474-2.647) on the raw dose scale. A t-test of the 4 differences, testing the hypothesis of no difference in IC50 values for BRCA2 proficient and deficient HCT116 cells yields t(3)=0.42, p = 0.71. Thus we fail to reject the null hypothesis and conclude that there is insufficient statistical evidence to suggest a difference in IC50 values for the BRCA2 proficient (WT, *BRCA2*$^{+/-}$) and deficient (B18, B46) genotypes of the HCT116 cell line.

(b) Drug sensitivity to BMH-21 was compared between BRCA2 proficient and deficient cells in DLD1. 3 independent WST-1 experiments were performed. The mean IC50 difference across the 3 experiments was 0.688 (raw dose scale ratio, 95%CI, 0.0578-8.18). A t-test of the 3 differences, testing the hypothesis of no difference in IC50 values for BRCA2 proficient and deficient DLD1 cells yields t(2)=-0.65, p = 0.58. Thus we fail to reject the null hypothesis and conclude that there is insufficient statistical evidence to suggest a difference in IC50 values for the BRCA2 proficient (WT, *BRAC2*$^{+/-}$) and deficient (*BRCA2*$^{-/-}$) genotypes of the DLD1 cell line.

(c) polR1B was effectively knocked down by siRNA, and knocking down polR1B decreased the expression of 45s pre-rRNA in U2OS. The expression of 45s pre-rRNA was analyzed by Taqman assay*** and Sybrgreen assay. Mean fold change in gene expression and 95%Cis are shown.

(d) BRCA2 knockout cells are highly sensitive to CX-3543 in DLD1. A graphical portrayal of the assay results across four independent experiments is shown with a smoother line for each cell genotype overlaid. There is strong statistical evidence of a difference in cell proliferation rates between the BRCA2 proficient (WT) and BRCA2 homozygous genotypes (BHOM) (F-test p<10-15) in the presence of CX-3543. Drug sensitivity of BRCA2 heterozygous cells (BHET) is not different from WT cells (p>0.2 by F-test across all drug concentrations tested).

(e and f) Cell viability was measured after polR1B and BRCA2 knockdown in HCT116 (e) and U2OS (f) by WST-1 assay. WST-1 intensity normalized to control was shown with mean value and 95%CI. Double knockdown for polR1B and BRCA2 differs significantly from any single knockdown in HCT116 (p<0.0001, omnibus test), but not in U2OS.

(g) 45S pre-rRNA level measured by RT-PCR after CX-5461, CX-3543 and BMH-21 treatment in WT and *BRCA2*$^{-/-}$ HCT116 cells. Drug incubation time was 2 hours. Fold change estimates and unadjusted 95% CIs of 45s pre-rRNA levels under drug treatment condition versus vehicle control are shown. qPCR was conducted using both Taqman and SYBR Green assays. Beta-actin was a loading control, and actinomycin treatment was a control for the reduction of 45s pre-rRNA.

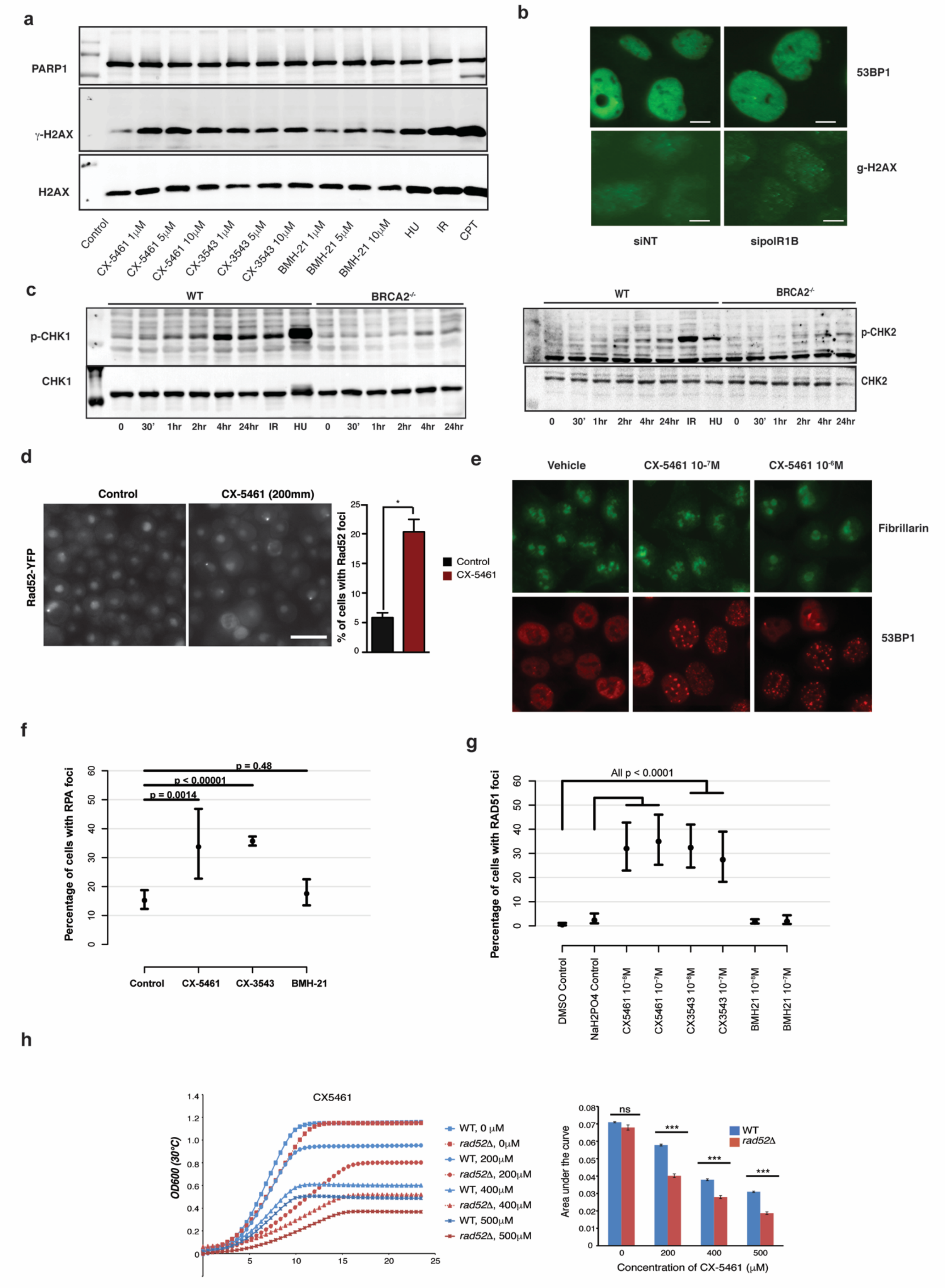

Supplementary Figure 4. (Relative to Figure 2). DNA damage was induced in cells with CX-5461 and CX-3543 treatment.

(a) The level of γ-H2AX increased upon CX-5461 and CX-3543 treatment for 24 hours in HCT116 via western blot analysis. The increased γ-H2AX level is not the result of cell death as indicated by the absence of PARP1 degradation. HU (5mM 2hr), IR (10Gy 1hr), and CPT (0.5 μM 24hr) were used as controls for the induction of DNA damage or apoptosis.
(b) polR1B knockdown in HCT116 did not increase γ-H2AX and 53BP1 foci formation.
(c) Chk1 and CHK2 phosphorylation increased upon CX-5461 treatment with 10^{-6} M CX-5461 in HCT116. HU (5mM 2hr) and IR (10Gy 1hr) were used as controls for the activation of DNA damage response. Cells were collected at different time points after drug treatment as indicated.
(d) CX-5461 treatment induced Rad52 foci formation in yeast. Left panel, Rad52-YFP tagged cells were treated with CX-5461 for 2hrs and imaged (Scale bar = 5μm; right panel, the percentage of cells with Rad52 foci was quantified comparing to the untreated controls (mean ± SEM; n=3 experiments) ($p<0.01$ for CX-5461 by t-test). Sharp RAD52-YPF focus indicates DNA damage; while diffused RAD52-YFP signal indicates no DNA damage.
(e) 53BP1 foci were observed in cells with intact nucleolus. WT HCT116 cells were treated with either vehicle or different concentrations of CX-5461 for 24hrs before immunofluorescence double staining with 53BP1 and fibrillarin. 53BP1 foci were significantly increased with 10^{-7}M CX-5461 (Figure 2c), while nucleolus was undamaged under this concentration. Scale bar=10μm. Experiments were repeated twice with representative images displayed.
(f and g) The percentage of cells with RPA foci (f), RAD51 Foci (g) upon CX-5461, CX-3543 and BMH-21 treatment in U2OS cells. RPA foci counting was repeated twice at 24hr upon 10^{-7}M CX-5461 treatment. RAD51 foci analysis was from at least three experiments upon 24hr of CX-5461 treatment. Bars represent 95% confidence interval. P-values were obtained from one-tail randomization tests. ***$p<0.001$.
(h) Yeast lacking RAD52 showed increased growth sensitivity to the CX-5461. Growth curves of isogenic wild type (WT) and rad52Δstrains with the CX-5461 at the indicated concentrations for 24hrs at 30°C. Each experiment was triplicated and averaged, and the area under each curve was measured (right panels) (mean ± SEM; n=3) (*** $p<0.0001$ t-test).

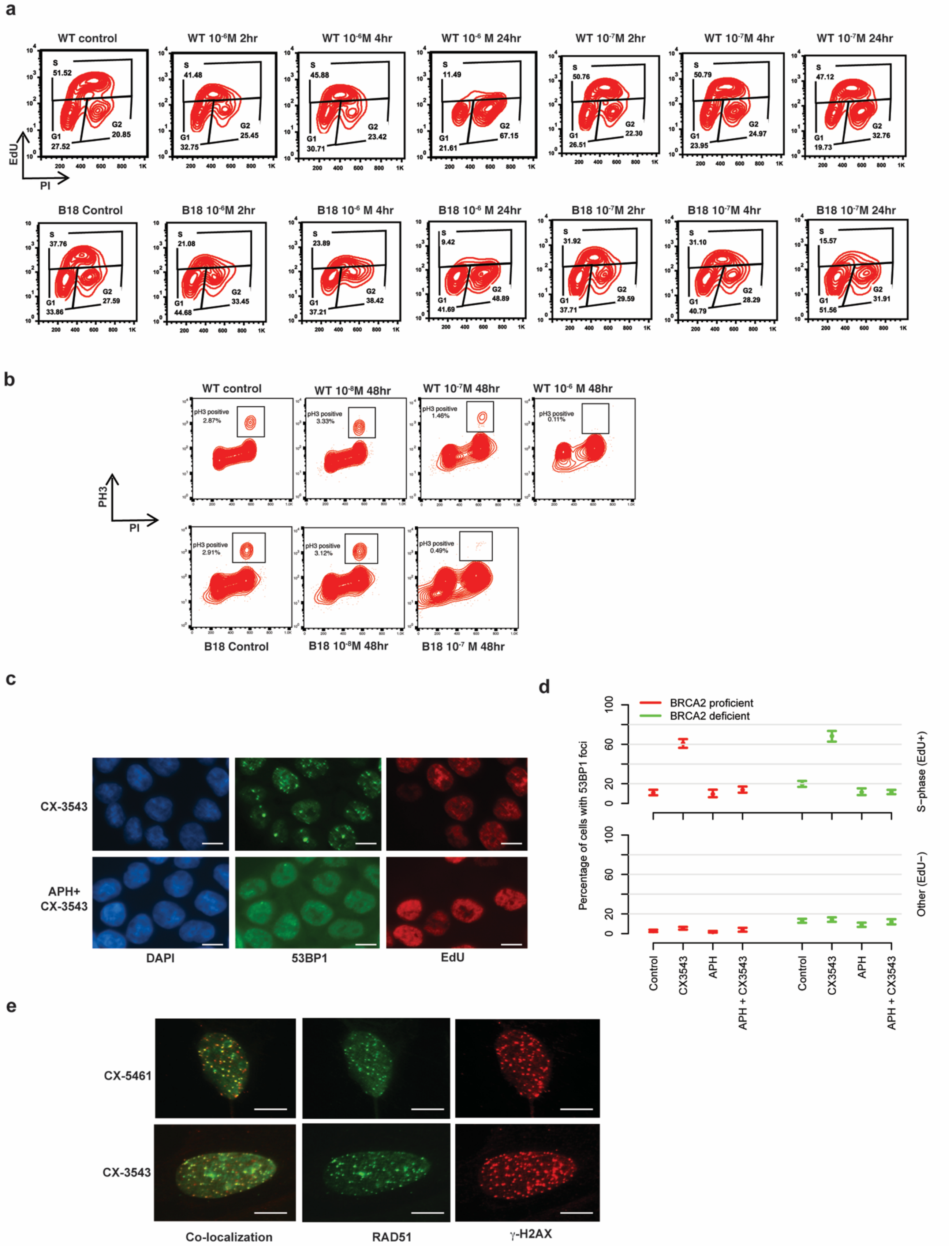

Supplementary Figure 5. (Relative to Figure 3, Figure 4) CX-5461 and CX-3543 induced DNA damage is replication-

dependent and its repair relies on BRCA2.

(a) Cell cycle analysis after CX-5461 treatment in HCT116. Representative picture of one experiment shown. The experiments were repeated 4 times. Percentage of cells in different cell cycle phase is summarized in supplementary Table 6.

(b) CX-5461 treatment blocks cells before mitosis. WT and *BRCA2*$^{-/-}$ HCT116 cells were treated with vehicle or CX-5461 at different concentration for 48 hours before subjected to dual PI and pH3 double staining and analyzed by flow cytometry. Experiments were repeated twice with similar results obtained.

(c) CX-3543 (10^{-7}M 1hr) induced 53BP1 foci enriched in S phase cells (positive with EdU labeling), and APH greatly suppressed CX-3543 induced DNA damage in HCT116. Cells were treated with EdU (20 μM) for 30min, then EdU was washed out and the cells were treated with CX-3543 (10^{-7} M) for 1 hour. For APH treatment, APH (5 μM) was added after EdU labelling and was treated for 2 hours before incubating with CX-3543 (10^{-7} M) for 1hr. Scale bar=10 μM.

(d) The percentage of 53BP1 foci positive cells within EdU positive and EdU negative population with or without APH was quantified. Experimental conditions were the same as stated in (C). Bars show the mean of three experiments (>100 cells each replica) and 95% confidence intervals.

(e) Co-localization of RAD51 foci and γ-H2AX foci upon CX-5461 and CX-3543 treatment in U2OS. Scale bar=10 μM. U2OS cells were treated with CX-5461 (10^{-6}M) for 24hr. Experiments were repeated three times.

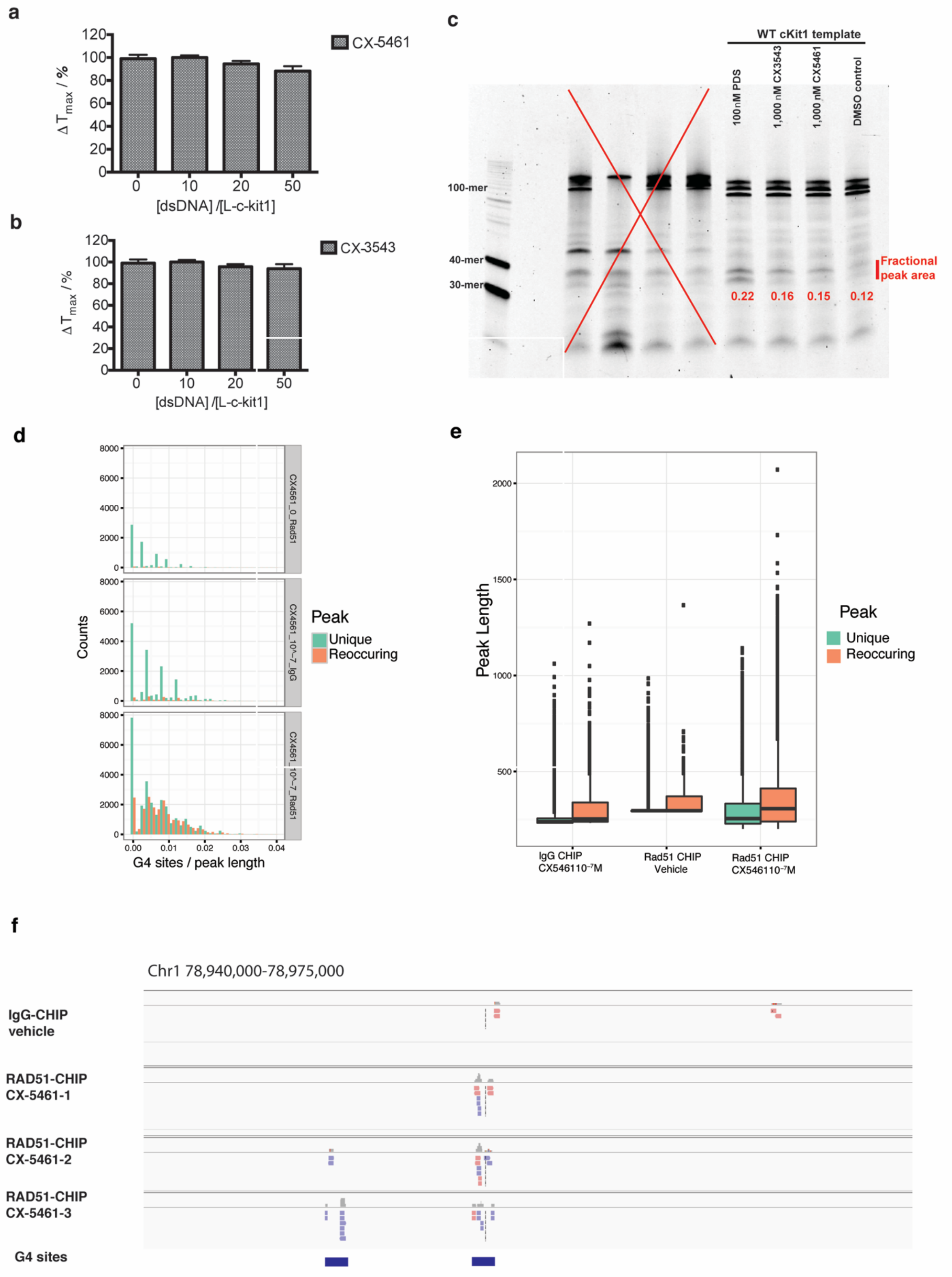

Supplementary Figure 6. (Relative to Figure 5,6) CX-5461 is a G4 stabilizer.

(a and b) Melting temperature change caused by CX-5461 and CX-3543 was not affected by unlabelled dsDNA. c-kit1 G-

quadruplex structure was pre-bound with CX-5461 (A) and CX-3543 (B), then a large amount of un-labelled dsDNA was added (up to 50 fold relative to the concentration of c-kit1).
(c) Original gel of Figure 5b. Unrelated lanes crossed out.
(d) The number of of G4 sites in unique and reoccurring peaks, normalized by peak length is shown. Peaks are identified from RAD51-ChIP. ChIP was carried out by using RAD51 antibody or IgG for immunoprecipitation with either vehicle or CX-5461 (10^{-7}M 24hours) treatment in U2OS.
(e) Peak length distributions in RAD51-ChIP. The treatment conditions are the same as Supplementary Fig. 6d. The peak length distributions were shown for unique and reoccurring peaks.
(f) A screen shot from IGV viewer showing the reads and peaks from RAD51-ChIP under CX5461 treatment (3 replicas) and IgG-ChIP. G4 sites on chromosome are indicated with blue rectangles.

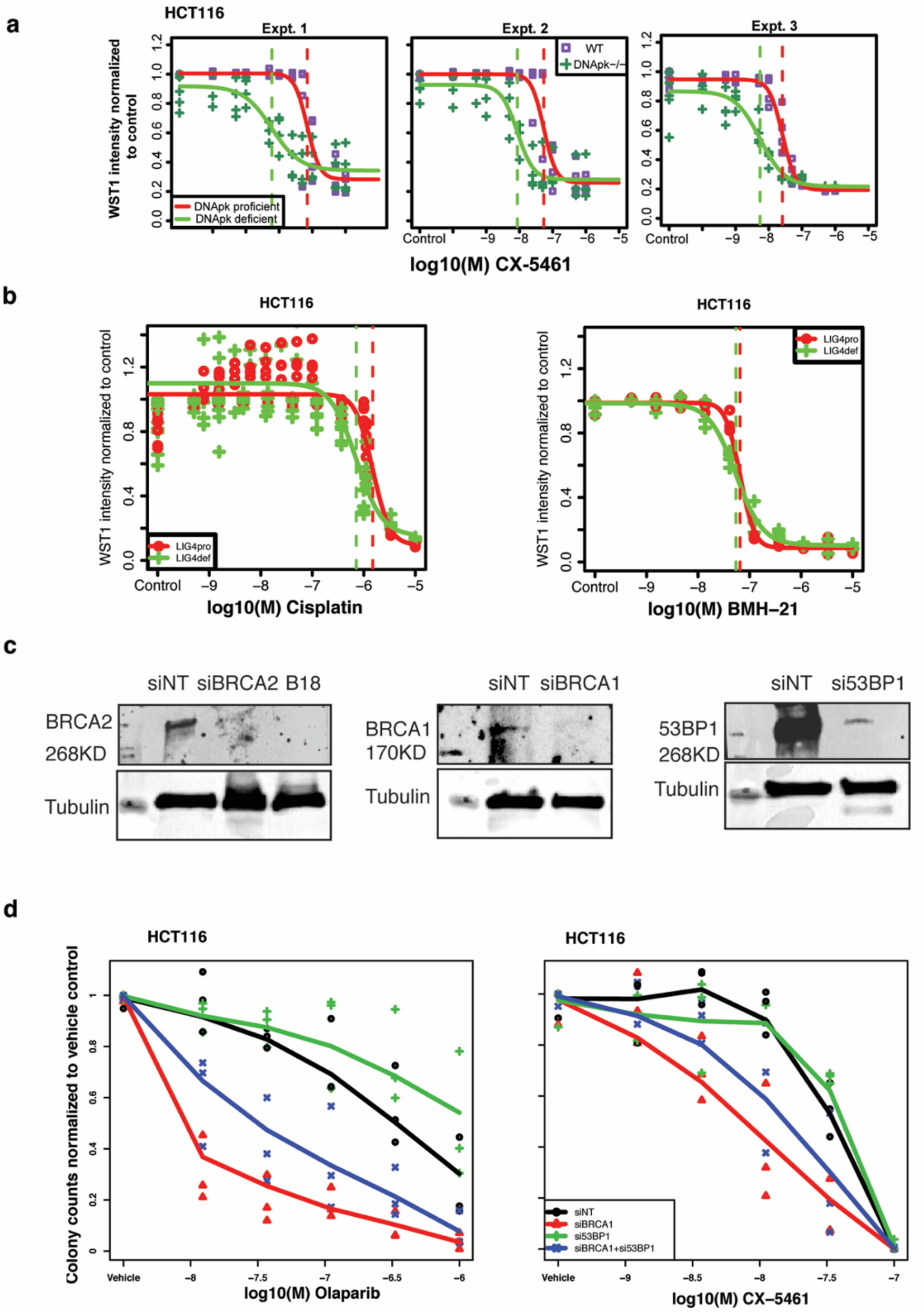

Supplementary Figure 7. (Relative to Figure 7) NHEJ pathway is involved in the repair of CX-5461 induced DNA damage.

(a) *PRKDC*$^{-/-}$ cells are more sensitive to CX-5461 comparing with PRKDC wild type cells in HCT116. All 3 WST-1 assay results are shown. The IC50 difference across the 3 experiments was 7.0192 down for PRKDC deficient relative to proficient (95%CI, 2.24-22.02) on the raw dose scale. A t-test of the 3 differences, testing the hypothesis of no difference in IC50 values for PRKDC proficient and deficient HCT116 cells yields $t(2)=7.33$, $p = 0.018$. Thus we reject the null hypothesis and conclude that there is sufficient statistical evidence to suggest a difference in IC50 values for the PRKDC proficient and deficient genotypes of the HCT116 cell line.

(b) Drug sensitivity results of *LIG4*$^{-/-}$ cells to cisplatin and BMH-21 comparing with *LIG4* wild type cells in HCT116. *LIG4*$^{-/-}$ cells are more sensitive to cisplatin than *LIG4*$^{+/+}$ cells, and the difference of IC50 is 2.08 ($p = 0.004$). IC50 difference for *LIG4*$^{-/-}$ cells vs. *LIG4*$^{+/+}$ cells is 1.2 down ($p= 0.008$) for BMH-21. Though statistically significant, such a small difference may be of little biological importance.

(c) The effective knockdown of BRCA1/2 and 53BP1 by siRNA transfection. Western blotting was carried out with HCT116 cells 4 days after knocking down with siRNA for non-targeting control or siRNA for specific gene. BRCA2 knock out clone

18 in HCT116 (B18) was used as a negative control for BRCA2 expression.

(d) 53BP1 knockdown cells showed similar sensitivity to CX-5461 as non-targeting control cells, but reduced the sensitivity of BRCA1 knockdown cells to CX-5461 (right panel). HCT116 cells were knocked down with either single or double siRNAs one day before plating for single cells and incubating with CX-5461. After 10 days, single cells grew into visible colonies and the number of colonies was counted. The effect of 53BP1 deficiency is negligible in the BRCA1 proficient genotype treated with CX-5461(F-test $p = 0.47$), but significant in BRCA1 deficient cells (F-test, $p = 0.008$). Left panel shows the rescue effect of 53BP1 deficiency in BRCA1 deficient cells to Olaparib sensitivity.

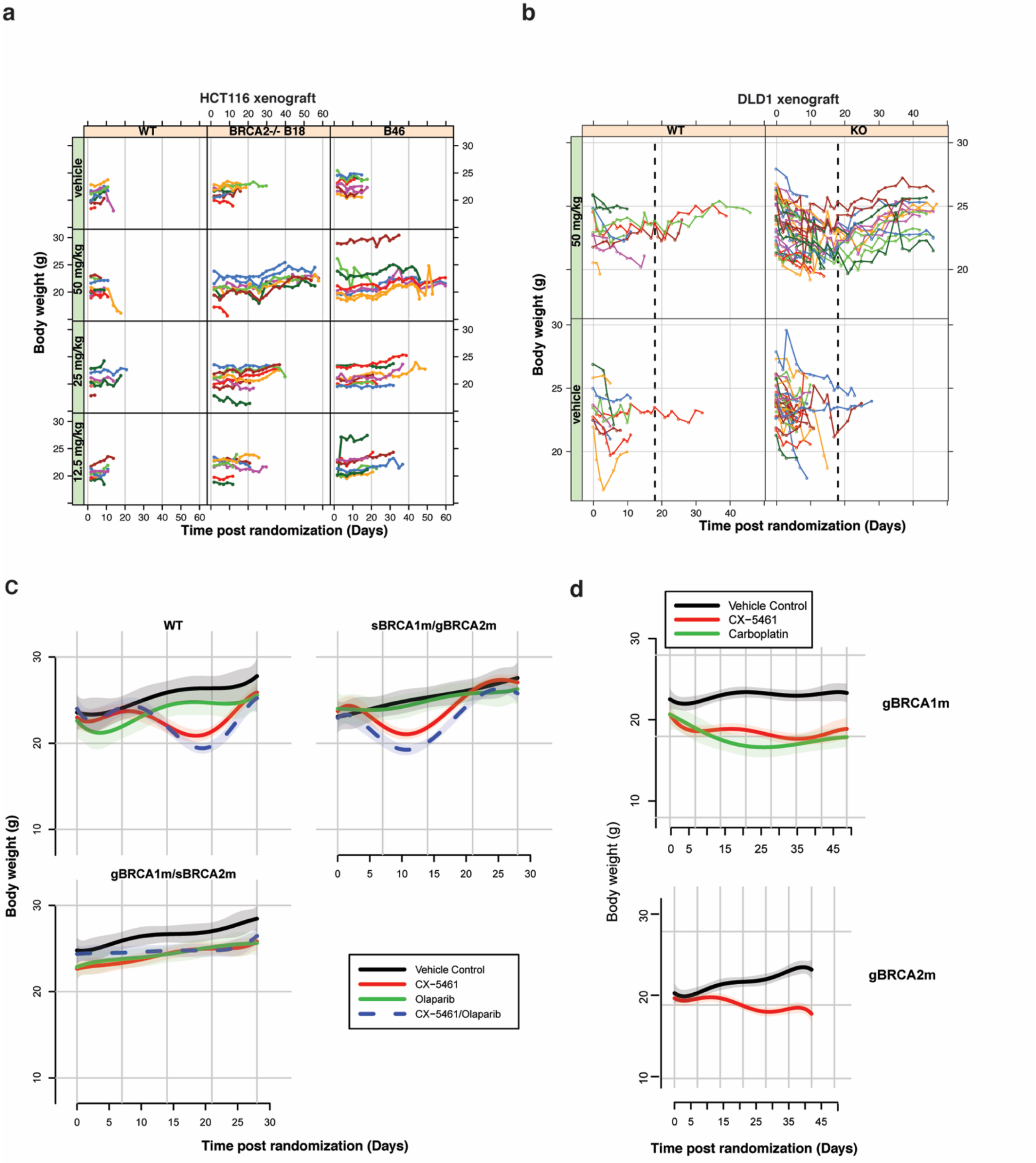

Supplementary Figure 8. (Relative to Figure 8) Body weight measurement of mice treated with CX-5461 in xenograft and PDX model.

(a) Body weight of mice transplanted with HCT116 WT, B18 or B46 cells.

(b) Body weight of xenograft experiments with DLD1 formed tumors.

(c) Body weight of mice administered intravenously with CX-5461 in model CTG-1019, CTG-0012 and CTG-0888. Solid lines represent the mean body weight with 95% CI (shown by shadow around solid lines).

(d) Body weight of mice in PDX model CFIB-NB02 (g*BRCA1*m) and CFIB-70620 (g*BRCA2*m). Solid lines represent the mean body weight with 95% CI (shown by shadow around solid lines).

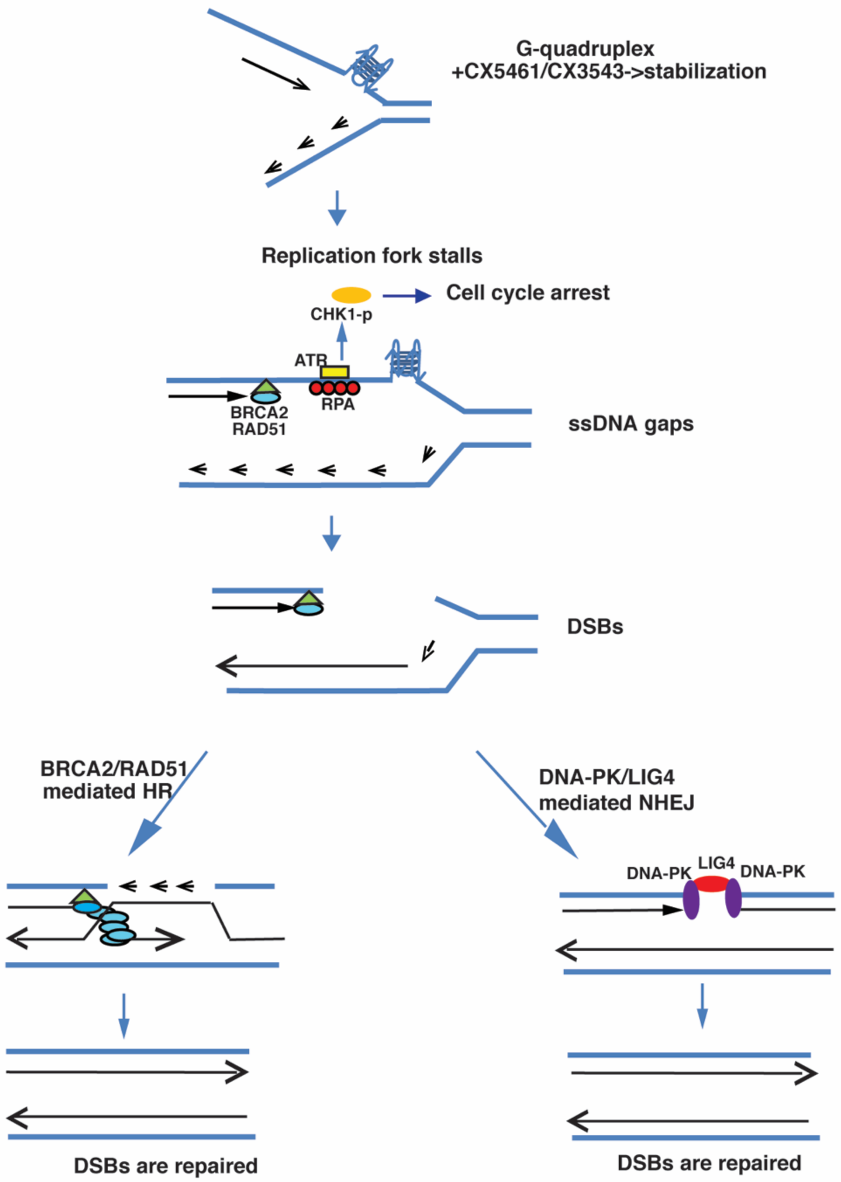

Supplementary Figure 9. A simplified model of replication block by CX-5461 or CX-3543 at G-quadruplex structure and the requirement of HR and NHEJ pathways in replication bypass and DNA damage repair.
See discussion for detailed explanation.

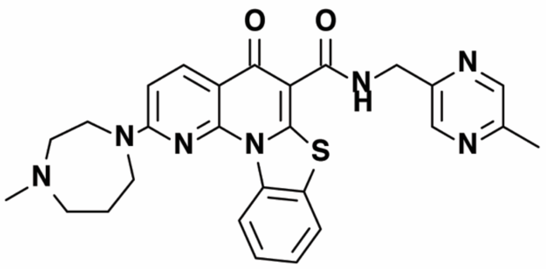

CX-5461

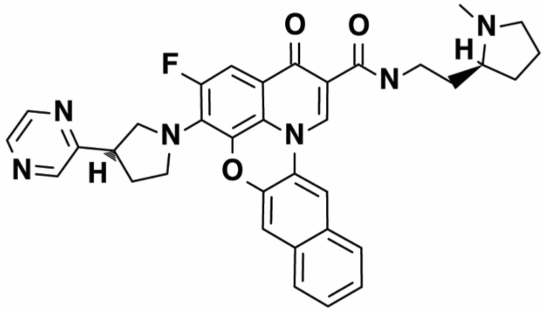

CX-3543

Supplementary Figure 10. Chemical structure of CX-5461 and CX-3543.

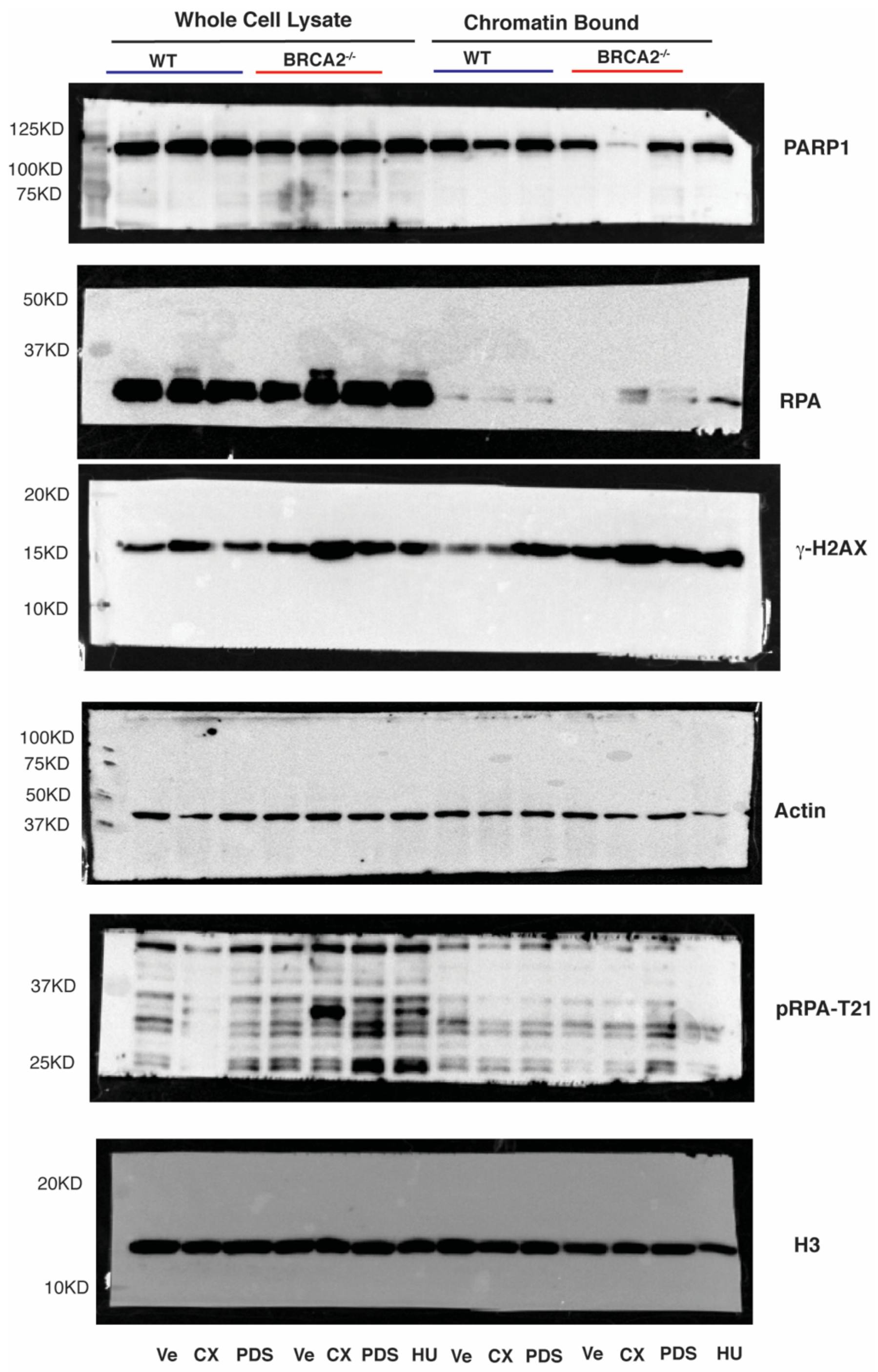

Supplementary Figure 11. Un-cropped western blotting images of Figure 4A.

Supplementary Table 1. Drugs and antibodies used in this study

Drugs tested for synthetic lethality against *BRCA2* knockout cells.

**Drug name**	**Drug target**	**Company and Catalogue number**
Olaparib	Parp1	Selleckchem, Cat. S1060
actinomycin	RNA polI and II	Sigma, Cat. A9415
ATM/ATR inhibitor	ATM/ATR	EMD Millipore, Cat. 118501
CHK1 inhibitor	CHK1	Tocris, TCS 2312
Chk2 inhibitor II	CHK2	EMD, Cat. 220486
ZM-447439	AurkB	Selleckbio, Cat. S1103
NU7441	DNA-PK	Tocris, Cat. 3712
MK2206	AKT	Chemie Tek, Cat. CT-MK2206
nutlin	MDM2	Sigma, Cat. N6287
CX-5461	RNA pol I	Selleckchem, S2684
Cycloheximide	translation	Sigma, Cat. C7698
LY 294002	PI3K	Tocris, LY 294002
Fludarabine	STAT1	Sigma, F2773
DZNep	EZH2	Victor E. Marquez's lab
QLT0267	ILK	QLT
CX-3543	RNA pol I	Adooq, A12380
BMH-21	RNA pol I	eMolecules

**Antibodies**	**Company**	**Cat. Number**	**Dilution**
phospho-Chk1S345	Cell Signal	2661S	1:1000
phospho-Chk2T68p	Cell Signal	2348L	1:1000
CHK1	Santa CruZ	SC-8408	1:1000
CHK2	Santa CruZ	SC-17747	1:1000
phospho-H2AX S139	Abcam	ab81299	1:6000 western<br>1:1000 IF
H2AX	Bethyl	A300-082A	1:6000
H3	Abcam	Ab1791	1:10,000
53BP1	BD Bioscience	612522	1:1000
RAD51	Santa CruZ	SC-8349	1:100
RPA2	Calbiochem	NA19L	1:1000 western<br>1:100 IF
RPA2-pT21	Abcam	ab61065	1:1000
PARP1	Santa CruZ	SC-8007	1:1000
BG4	Shankar Balasubramanian's lab		1:200

Supplementary Table 2. List of *C. elegans* mutants tested for sensitivity to 100 μM CX-5461 using a chronic exposure assay. + indicates that the mutant was sensitive to CX-5461 compared to carrier alone in 3/3 assays.

Human homolog	Gene	*allele*	CX5461 sensitivity	Function
**DNA damage response**				
iASPP	*ape-1*	*ok1045*	-	DNA Damage Checkpoint
TRIP13	*pch-2*	*tm1458*	-	DNA Damage Checkpoint
HUS1	*hus-1*	*op241*	-	DNA Damage Checkpoint
TP53	*cep-1*	*gk138*	-	DNA Damage Checkpoint
ATM	***atm-1***	***tm5029***	**+**	**DNA Damage Checkpoint**
UNG	*ung-1*	*ok3593*	-	DNA glycosylase
TP53BP1	*hsr-9*	*ok729*	-	DNA Repair
GEN1	*gen-1*	*tm2940*	-	Endonuclease
SLX1A	*slx-1*	*tm2644*	-	Endonuclease
ERCC4	*xpf-1*	*tm2842*	-	Endonuclease
MUS81	***mus-81***	***tm1937***	**+**	**Endonuclease**
FAN1	*fan-1*	*tm423*	-	Endonuclease
EXD3	*mut-7*	*pk204*	-	Exonuclease
POLQ	***polq-1***	***tm2026***	**+**	**DNA polymerase**
FANCD2	*fcd-2*	*tm1298*	-	FA pathway
ATRX	*xnp-1*	*tm678*	-	Helicase
RECQL	*rcq-1*	*tm3870*	-	Helicase
RECQL5	*rcq-5*	*tm424*	-	Helicase
RTEL1	***rtel-1***	***tm1866***	**+**	**Helicase**
HELQ	***helq-1***	***tm2134***	**+**	**Helicase**
BLM	*him-6*	*ok412*	-	Helicase
FANCJ	*dog-1*	*gk10*	-	Helicase
WRN	*wrn-1*	*gk99*	-	Helicase
BARD1	*brd-1*	*gk297*	-	HR
BRCA1	*brc-1*	*ok1261*	-	HR
RAD51D	***rfs-1***	***ok1572***	**+**	**HR**
BRCA2	***brc-2***	***ok1629/+***	**+**	**HR**
MLH1	*mlh-1*	*ok516*	-	MMR
ERCC6	*csb-1*	*ok2335*	-	NER
LIG4	*lig-4*	*ok716*	-	NHEJ
XRCC5	*cku-80*	*ok861*	-	NHEJ
**Chromatin and transcription**				
NCAPD2	*dpy-28*	*y1*	-	Chromatin
CHD3	*chd-3*	*ok1651*	-	Chromatin remodeling
CHD7	*chd-7*	*gk306*	-	Chromatin remodeling
HAT1	*hat-1*	*ok1265*	-	Chromatin remodeling
L3MBTL2	*mbtr-1*	*n4775*	-	Chromatin remodeling
MTA1	*lin-40*	*ku285*	-	Chromatin remodeling
UTY	*jmjd-3.1*	*ok384*	-	Chromatin remodeling
HDAC4	*hda-4*	*ok518*	-	Chromatin remodeling
RCOR3	*spr-1*	*ok2144*	-	Chromatin remodeling

HDAC3	*hda-3*	*ok1991*	-	Chromatin remodeling
ARID3A	*cfi-1*	*os122*	-	Chromatin remodeling
HDAC2	*hda-2*	*ok1479*	-	Chromatin remodeling
SIRT1	*sir-2.1*	*ok434*	-	Chromatin remodeling
CREBBP	***cbp-1***	***ku258***	**+**	**Chromatin remodeling**
MTA1	*egl-27*	*n170*	-	Chromatin remodeling
CHD4	***let-418***	***n3536***	**+**	**Chromatin remodeling**
SMEK1	***smk-1***	***mn156***	**+**	**Chromatin remodeling**
H1F0	*hil-1*	*ok229*	-	Histone
HIST1H1B	*his-24*	*ok1024*	-	Histone
H2AFZ	***htz-1***	***ok3100/+***	**+**	**Histone**
RFX2	*daf-19*	*m86*	-	Transcription
SIN3B	*sin-3*	*kc565*	-	Transcription
**PAR metabolism**				
PARP1	*parp-1*	*ok988*	-	PARP
PARG	***parg-1***	***gk120***	**+**	**PARG**
PARP2	*parp-2*	*tm3401*	-	PARP
TNKS	***tank-1***	***ok446***	**+**	**Tankyrase**
PARG	***parg-2***	***ok980***	**+**	**PARG**
**Replication**				
TIPIN	*csm-3*	*tm4638*	-	
GINS3	*psf-3*	*ok2828*	-	
MCM4	*mcm-4*	*n3809*	-	
SBF2	*mtm-5*	*ok469*	-	
**Ubiquitin metabolism**				
CUL1	*cul-6*	*ok1614*	-	
UBE2A	*ubc-1*	*gk14*	-	
UBE2F	*ubc-12*	*ok688*	-	
UBE2Q1	*ubc-25*	*ok1732*	-	
UBE3B	*oxi-1*	*ok1217*	-	
AMFR	*hrdl-1*	*gk28*	-	
USP7	*math-33*	*ok2974*	-	
**Spindle Checkpoint**				
BUB1B	*san-1*	*ok1580*	-	
MAD2L1	*mdf-2*	*vc15*	-	
**RNA Binding**				
QKI	***gld-1***	***op236***	**+**	***cep-1/p53*** **regulator**
UPF2	***smg-3***	***ma117***	**+**	**Nonsense mediated decay**
**Other**				
VPS11	*vps-11*	*ok1664*	-	
DAB2IP	*gap-2*	*tm748*	-	
CSNK1D	*kin-20*	*ok505*	-	
ANKLE1	*lem-3*	*mn155*	-	

C14orf164	*zhp-3*	*ok1993*	-
SHC1	*shc-1*	*ok198*	-
TERT	*trt-1*	*ok410*	-
RSBN1L	*dpy-21*	*e428*	-
CDC14A	*cdc-14*	*he141*	-
PFDN5	*pfd-5*	*gk706*	-

Supplementary Table 3. BRCA status of PDX Models

ID	BRCA Status	Characterization
**CTG-1019**	gBRCA1m (K1183R; P871L)<br><br>WT BRCA2	The patient derived xenograft (PDX) tumor was derived from a 53 year old Caucasian woman with Stage IV TNBC. Prior to the PDX tumor harvesting, the patient was treated with Cyclophosphamide/ Doxorubicin/Paclitaxel for duration of 28 months. Upon treatment failure, she was put on Docetaxel/ Capecitabine for 10 months. The patient was then placed on treatments with Capecitabine, Eribulin and Doxil (each as a single agent) with progression of her cancer soon after. Next Generation Sequencing (NGS) revealed BRCA1 mutation (non-deleterious).
**CTG-0012**	sBRCA1m (Y978*)<br><br>gBRCA2m (N372H)	The PDX tumor was derived from a 36 year old Caucasian woman with Stage IV TNBC. Prior to the PDX tumor harvesting, she was treated with Docetaxel for a duration of 12 months before her disease progressed. The patient was subsequently treated with Capecitabine/Bevacizumab and Vinorelbine/ Bevacizumab but failed both treatments. NGS Data revealed a deleterious somatic mutation in BRCA1 as well as a germline mutation in BRCA2.
**CTG-0888**	sBRCA2m (X3030X–frameshift)<br><br>gBRCA1m (K1183R; S1634G; P871L; E1038G)	The PDX tumor was derived from a 43 year old Caucasian woman with Stage III TNBC. Prior to PDX tumor harvesting, she was treated with Cyclophosphamide/Doxorubicin/Paclitaxel for a duration of 9 months. NGS data revealed a deleterious somatic mutation in BRCA2 and germline mutations in BRCA1
**CFIB-NB02**	gBRCA1m (c2477C>A)	TNBC patient with germline BRCA1 exon 11 missense variant c2477C>A; was diagnosed at 39; with family history of ovary cancer at age 37; heavily pretreated in phase I clinic (including prior platinum).
**CFIB-70620**	gBRCA2m (c793+6T>C)	35 year TNBC patient with BRCA2 variant; germline BRCA2 - EXON09 c793+6T>C; from somewhere in Asia; multiple family members with “breast lumps”; somatic TP53 mutation; sample is from post-neoadjuvant FEC/D.; subsequently had minor response to gemcitabine/cisplatin; very aggressive disease died within 1 year of breast surgery.

Supplementary Table 4. Summary of breast cancer cell lines used in Figure 7d

Cell Line Name	BMH21 log10. IC50.	CX3543 log10. IC50.	CX5461. log10. IC50.	Olaparib .log10. IC50.	Cisplatin. log10. IC50.	subtype intrinsic	Genotype of BRCA1/2, RAD51	Mutation in DNA.damage. genes
MDA-MB-436	-6.82	-9.30	-9.30	-7.42	NA	CL	BRCA1 homo	ATM, ARID1B, BRCC3, FANCI
CAL-148	-6.93	-8.21	-7.98	-3.30	NA	HER2	RAD50 het	PTEN het,
600MPE	-7.01	-8.09	-7.97	-5.76	NA	LuminalA		
CAL-51	-6.85	-7.61	-7.67	-5.34	-5.72	CL	BRCA2 het, RAD51C het	ARID1A, PTEN homo, ATM het
HCC1187	-7.66	-7.73	-7.59	-6.60	-5.32	Basal		BAP1 homo
SUM159PT	-6.60	-7.49	-7.54	-5.58	NA	CL		MLH1
OCUB-M	-6.58	-7.65	-7.52	-5.43	-5.19	LuminalB		BARD1, HELQ, MLH1
HBL-100	-6.90	-7.43	-7.49	-3.30	NA	CL		ATM, PTEN
MDA-MB-231	-7.21	-7.32	-7.40	-5.48	-5.46	CL		ATM het, ATR, BRAF, MLH1
SW527	-7.10	-7.56	-7.38	-5.49	NA	Basal		BARD1 low expression
MDA-MB-468	-6.42	-7.16	-7.28	-5.43	-5.72	Basal	BRCA2 het	ATM, BLM homo, FANCI het, PTEN homo
EFM-19	-6.40	-8.53	-7.26	-3.30	-5.26	LuminalA		ATR homo
CAL-120	-6.76	-6.85	-7.25	-3.30	-5.34	CL		ATM, FANCG homo
HCC1806	-7.42	-7.30	-7.24	-5.94	-5.70	Basal	BRCA1 homo	PTEN loss
HCC1954	-6.57	-7.41	-7.21	-4.83	-5.27	HER2	BRCA1 het	FANCL down expression
HDQ-P1	-7.04	-7.12	-7.20	-5.10	NA	Normal		POlR1A homo, POLR1B, RAD17 het
BT-20	-6.85	-7.02	-7.14	-5.51	-5.27	Basal	BRCA2 mutation	ATM , MLH1 mutation
MDA-MB-330	-6.06	-6.09	-7.13	-3.30	NA	LuminalA		ATM, ATR, MLH1, FANCD2
HCC1395	-6.58	-7.10	-7.13	-5.93	-5.60	CL	BRCA2 het	ATRX homo
SK-BR-7	-6.57	-7.17	-7.13	-3.30	NA	CL		ATM, ATR
MCF-7	-6.87	-6.99	-7.11	-5.45	-5.39	LuminalA		
KPL-1	-6.79	-7.06	-7.07	-6.00	NA	LuminalB		
MCF-7/LY2	-6.62	-7.14	-7.05	-3.30	NA	LuminalA		
HCC38	-6.79	-7.10	-7.05	-5.50	-5.31	Basal	BRCA1 low expression	
HCC1143	-6.68	-6.99	-7.05	-3.30	-5.41	Basal		PTEN null
Hs 578T	-6.40	-7.13	-7.03	-3.30	-5.62	CL		ATR, MLH1 mutation
ZR-75-1	-6.42	-6.96	-6.97	-5.75	NA	LuminalA		ATR , PTEN, MLH1
MDA-MB-361	-6.31	-7.05	-6.96	-5.45	-5.54	HER2		MSH2
SK-BR-3	-6.28	-6.90	-6.94	-3.30	NA	HER2	BRCA1 loss expression	
BT-549	-6.78	-6.89	-6.88	-5.10	-5.32	CL		
T-47D	-6.24	-7.17	-6.85	-3.30	-5.26	LuminalB		ATM, ARID1A
CAMA-1	-6.43	-6.64	-6.82	-3.30	-5.74	LuminalB		MLH1

AU565	-6.32	-6.88	-6.78	-3.30	-5.65	HER2		
HCC1937	-6.74	-6.67	-6.74	-5.33	-5.27	Basal	BRCA1 homo	
SK-BR-5	-6.54	-6.78	-6.72	-5.10	NA	LuminalB		ATM
Evsa-T	-6.43	-6.59	-6.61	-3.30	-5.61	HER2		ATR, MLH, PTE
JIMT-1	-6.66	-6.61	-6.55	-3.30	NA	Basal		ATR, MLH1
MFM-223	-6.43	-6.56	-6.50	-5.38	-5.36	LuminalB		ATM , PTEN
HCC2185	-6.21	-5.50	-6.30	-5.01	NA	LuminalB		MLH1
UACC-893	-6.33	-5.62	-6.22	-4.61	-5.49	HER2		
BT-474	-6.25	-6.64	-5.73	-3.30	-5.15	LuminalA	BRCA2 nonsense mutation	
HCC70	-6.10	-6.10	-5.71	-3.30	-5.58	Basal		MLH1, PTEN
HCC3153	-6.17	-3.30	-5.71	-5.66	NA	Basal		ATM
MDA-MB-175-VII	-6.14	-5.65	-5.49	-5.53	-5.37	LuminalA		
HCC1419	-6.00	-5.72	-5.12	-3.30	-5.47	LuminalA		
MDA-MB-157	-6.35	-3.94	-5.07	-3.30	-5.32	CL		
HCC1428	-6.01	-3.30	-3.30	-5.43	NA	LuminalB		ATM, MLH1
MDA-MB-134-VI	-6.13	-3.30	-3.30	-3.30	NA	LuminalB		ARID1A
HCC202	-6.30	-3.30	-3.30	-5.92	NA	HER2		ATM, MLH1
MX-1	-5.91	-3.30	-3.30	-3.30	NA	Basal		ATM, ATR

Disease subtype information is described in published paper (Marcotte, R. et al., 2016) and Cosmic (http://cancer.sanger.ac.uk/cell_lines). Cisplatin sensitivity data are extracted from Genomics of Drug Sensitivity in Cancer program (http://www.cancerrxgene.org/translation/Drug/1005#t_IC50_1005).

Supplementary Table 5. Percentage of apoptotic and dead cells after CX-5461 treatment for 72 hours in WT and *BRCA2*$^{-/-}$ HCT116 cells evaluated by FACS.

Apoptotic cell population

ID	Contrast Name	Difference in apoptosis rate (percent): Estimate	Difference in apoptosis rate (percent): 95% lower confidence limit	Difference in apoptosis rate (percent): 95% upper confidence limit	Z value	p-value	Benjamini-Hochberg adjusted p-value
1	Bd Control v Bp Control	1.32%	-0.66%	3.31%	1.31	0.19	0.28
2	Bd CX5461_10^-8M v Bp CX5461_10^-8M	4.54%	2.56%	6.53%	4.49	7.19E-06	1.64E-05
3	Bd CX5461_10^-7M v Bp CX5461_10^-7M	9.96%	7.97%	11.94%	9.84	7.62E-23	1.22E-21
4	Bd CX5461_10^-6M v Bp CX5461_10^-6M	8.59%	6.37%	10.81%	7.59	3.16E-14	1.26E-13
5	Bp CX5461_10^-8M v Bp Control	0.05%	-1.93%	2.04%	0.05	0.96	0.96
6	Bp CX5461_10^-7M v Bp Control	0.91%	-1.07%	2.90%	0.90	0.37	0.49
7	Bp CX5461_10^-6M v Bp Control	1.53%	-0.60%	3.65%	1.41	0.16	0.28
8	Bp CX5461_10^-7M v Bp CX5461_10^-8M	0.86%	-1.12%	2.84%	0.85	0.40	0.49
9	Bp CX5461_10^-6M v Bp CX5461_10^-8M	1.47%	-0.65%	3.60%	1.36	0.17	0.28
10	Bp CX5461_10^-6M v Bp CX5461_10^-7M	0.61%	-1.51%	2.74%	0.57	0.57	0.61
11	Bd CX5461_10^-8M v Bd Control	3.27%	1.29%	5.26%	3.23	0.0012	0.0024
12	Bd CX5461_10^-7M v Bd Control	9.55%	7.56%	11.53%	9.43	3.94E-21	3.15E-20

13	Bd CX5461_10^-6M v Bd Control	8.79%	6.67%	10.92%	8.12	4.78E-16	2.55E-15
14	Bd CX5461_10^-7M v Bd CX5461_10^-8M	6.28%	4.29%	8.26%	6.20	5.60E-10	1.79E-09
15	Bd CX5461_10^-6M v Bd CX5461_10^-8M	5.52%	3.40%	7.64%	5.10	3.46E-07	9.22E-07
16	Bd CX5461_10^-6M v Bd CX5461_10^-7M	-0.75%	-2.88%	1.37%	-0.70	0.49	0.56

Dead Cells population

ID	Contrast Name	Difference in dead or late apoptosis rate (percent): Estimate	Difference in dead or late apoptosis rate (percent): 95% lower confidence limit	Difference in dead or late apoptosis rate (percent): 95% upper confidence limit	Z value	p-value	Benjamini-Hochberg adjusted p-value
1	Bd Control v Bp Control	5.26%	1.89%	8.63%	3.06	0.0022	0.0051
2	Bd CX5461_10^-8M v Bp CX5461_10^-8M	3.45%	-0.18%	7.07%	1.87	0.062	0.111
3	Bd CX5461_10^-7M v Bp CX5461_10^-7M	11.08%	7.45%	14.70%	5.99	2.06E-09	3.30E-08
4	Bd CX5461_10^-6M v Bp CX5461_10^-6M	8.30%	4.53%	12.06%	4.32	1.58E-05	1.26E-04
5	Bp CX5461_10^-8M v Bp Control	1.87%	-1.50%	5.24%	1.09	0.28	0.42
6	Bp CX5461_10^-7M v Bp Control	1.69%	-1.68%	5.06%	0.99	0.32	0.42
7	Bp CX5461_10^-6M v Bp Control	3.62%	0.00%	7.25%	1.96	0.05	0.10
8	Bp CX5461_10^-7M v Bp CX5461_10^-8M	-0.17%	-3.54%	3.20%	-0.10	0.92	0.98
9	Bp CX5461_10^-6M v Bp CX5461_10^-8M	1.76%	-1.87%	5.38%	0.95	0.34	0.42
10	Bp CX5461_10^-6M v Bp CX5461_10^-7M	1.93%	-1.69%	5.55%	1.04	0.30	0.42
11	Bd CX5461_10^-8M v Bd Control	0.06%	-3.57%	3.68%	0.03	0.98	0.98
12	Bd CX5461_10^-7M v Bd Control	7.51%	3.89%	11.14%	4.07	4.79E-05	2.55E-04
13	Bd CX5461_10^-6M v Bd Control	6.66%	3.04%	10.29%	3.61	3.11E-04	9.96E-04
14	Bd CX5461_10^-7M v Bd CX5461_10^-8M	7.46%	3.69%	11.22%	3.88	1.04E-04	4.17E-04
15	Bd CX5461_10^-6M v Bd CX5461_10^-8M	6.61%	2.84%	10.37%	3.44	0.0006	0.0016
16	Bd CX5461_10^-6M v Bd CX5461_10^-7M	-0.85%	-4.62%	2.92%	-0.44	0.66	0.75

Bp: HCT116 BRCA2 proficient cells (Wild type)
Bd: HCT116 BRCA2 deficient cells (B18 $BRCA2^{-/-}$)
Green color indicates statistically significant difference in pairwise comparison.

Supplementary Table 6. Percentage of cells in different cell cycle stages with CX-5461 treatment in WT and *BRCA2*$^{-/-}$ HCT116 cells.

G1 phase

ContrID	Contrast Name	Difference in percent of cells in G1 phase: Estimate	Difference in percent of cells in G1 phase: 95% lower confidence limit	Difference in percent of cells in G1 phase: 95% upper confidence limit	Z value	p-value	Benjamini-Hochberg adjusted p-value
1	Bp CX5461 10^-7M 2hr v Bp Control	-4.0	-8.4	0.4	-1.80	0.072	0.18
2	Bp CX5461 10^-7M 4hr v Bp Control	-1.8	-6.1	2.6	-0.80	0.43	0.58
3	Bp CX5461 10^-7M 24hr v Bp Control	-8.5	-12.9	-4.1	-3.77	0.00016	0.0016
4	Bp CX5461 10^-7M 4hr v Bp CX5461 10^-7M 2hr	2.3	-3.1	7.7	0.82	0.41	0.58
5	Bp CX5461 10^-7M 24hr v Bp CX5461 10^-7M 2hr	-4.4	-9.5	0.6	-1.73	0.084	0.19
6	Bp CX5461 10^-7M 24hr v Bp CX5461 10^-7M 4hr	-6.7	-12.1	-1.3	-2.43	0.015	0.050
7	Bp CX5461 10^-6M 2hr v Bp Control	2.8	-1.6	7.2	1.24	0.21	0.38
8	Bp CX5461 10^-6M 4hr v Bp Control	0.4	-3.9	4.8	0.19	0.85	0.95
9	Bp CX5461 10^-6M 24hr v Bp Control	-7.3	-11.7	-2.9	-3.24	0.0012	0.0060
10	Bp CX5461 10^-6M 4hr v Bp CX5461 10^-6M 2hr	-2.4	-7.8	3.0	-0.86	0.39	0.58
11	Bp CX5461 10^-6M 24hr v Bp CX5461 10^-6M 2hr	-10.1	-15.1	-5.0	-3.93	8.60E-05	0.0013
12	Bp CX5461 10^-6M 24hr v Bp CX5461 10^-6M 4hr	-7.7	-13.1	-2.3	-2.79	0.0052	0.022
13	Bp CX5461 10^-6M 2hr v Bp CX5461 10^-7M 2hr	6.8	1.8	11.9	2.66	0.0078	0.029
14	Bp CX5461 10^-6M 4hr v Bp CX5461 10^-7M 4hr	2.2	-2.8	7.2	0.85	0.39	0.58
15	Bp CX5461 10^-6M 24hr v Bp CX5461 10^-7M 24hr	1.2	-3.8	6.2	0.46	0.64	0.84
16	Bd CX5461 10^-7M 2hr v Bd Control	0.2	-4.4	4.8	0.08	0.94	0.95
17	Bd CX5461 10^-7M 4hr v Bd Control	-0.1	-4.7	4.5	-0.06	0.95	0.95
18	Bd CX5461 10^-7M 24hr v Bd Control	9.5	4.8	14.1	4.01	6.13E-05	0.0013
19	Bd CX5461 10^-7M 4hr v Bd CX5461 10^-7M 2hr	-0.3	-5.7	5.1	-0.12	0.91	0.95
20	Bd CX5461 10^-7M 24hr v Bd CX5461 10^-7M 2hr	9.3	4.2	14.3	3.62	0.00030	0.0023
21	Bd CX5461 10^-7M 24hr v Bd CX5461 10^-7M 4hr	9.6	4.2	15.0	3.48	0.00050	0.0030
22	Bd CX5461 10^-6M 2hr v Bd Control	3.8	-0.8	8.4	1.60	0.11	0.23
23	Bd CX5461 10^-6M 4hr v Bd Control	0.7	-3.9	5.3	0.31	0.76	0.90

24	Bd CX5461 10^-6M 24hr v Bd Control	4.5	-0.1	9.1	1.90	0.057	0.16
25	Bd CX5461 10^-6M 4hr v Bd CX5461 10^-6M 2hr	-3.1	-8.5	2.3	-1.11	0.27	0.44
26	Bd CX5461 10^-6M 24hr v Bd CX5461 10^-6M 2hr	0.7	-4.3	5.7	0.28	0.78	0.90
27	Bd CX5461 10^-6M 24hr v Bd CX5461 10^-6M 4hr	3.8	-1.6	9.2	1.37	0.17	0.32
28	Bd CX5461 10^-6M 2hr v Bd CX5461 10^-7M 2hr	3.6	-1.4	8.6	1.40	0.16	0.32
29	Bd CX5461 10^-6M 4hr v Bd CX5461 10^-7M 4hr	0.9	-4.2	5.9	0.34	0.74	0.90
30	Bd CX5461 10^-6M 24hr v Bd CX5461 10^-7M 24hr	-5.0	-10.0	0.1	-1.93	0.053	0.16

S phase

ContrID	Contrast Name	Difference in percent of cells in S phase: Estimate	Difference in percent of cells in S phase: 95% lower confidence limit	Difference in percent of cells in S phase: 95% upper confidence limit	Z value	p-value	Benjamini-Hochberg adjusted p-value
1	Bp CX5461 10^-7M 2hr v Bp Control	1.65	-2.78	6.08	0.73	0.47	0.60
2	Bp CX5461 10^-7M 4hr v Bp Control	1.59	-2.80	5.98	0.71	0.48	0.60
3	Bp CX5461 10^-7M 24hr v Bp Control	0.24	-4.19	4.67	0.11	0.91	0.95
4	Bp CX5461 10^-7M 4hr v Bp CX5461 10^-7M 2hr	-0.06	-5.50	5.38	-0.02	0.98	0.98
5	Bp CX5461 10^-7M 24hr v Bp CX5461 10^-7M 2hr	-1.40	-6.46	3.66	-0.54	0.59	0.68
6	Bp CX5461 10^-7M 24hr v Bp CX5461 10^-7M 4hr	-1.34	-6.78	4.09	-0.48	0.63	0.70
7	Bp CX5461 10^-6M 2hr v Bp Control	-7.06	-11.49	-2.63	-3.12	0.0018	0.0036
8	Bp CX5461 10^-6M 4hr v Bp Control	-1.95	-6.34	2.44	-0.87	0.38	0.52
9	Bp CX5461 10^-6M 24hr v Bp Control	-33.76	-38.19	-29.33	-14.93	1.97E-50	5.91E-49
10	Bp CX5461 10^-6M 4hr v Bp CX5461 10^-6M 2hr	5.11	-0.32	10.55	1.84	0.065	0.109
11	Bp CX5461 10^-6M 24hr v Bp CX5461 10^-6M 2hr	-26.70	-31.76	-21.64	-10.34	4.44E-25	3.33E-24
12	Bp CX5461 10^-6M 24hr v Bp CX5461 10^-6M 4hr	-31.81	-37.25	-26.38	-11.47	1.88E-30	1.88E-29
13	Bp CX5461 10^-6M 2hr v Bp CX5461 10^-7M 2hr	-8.71	-13.77	-3.65	-3.37	0.00074	0.00171
14	Bp CX5461 10^-6M 4hr v Bp CX5461 10^-7M 4hr	-3.54	-8.60	1.52	-1.37	0.17	0.24
15	Bp CX5461 10^-6M 24hr v Bp CX5461 10^-7M 24hr	-34.01	-39.07	-28.95	-13.18	1.22E-39	1.83E-38
16	Bd CX5461 10^-7M 2hr v Bd Control	-3.54	-8.19	1.12	-1.49	0.14	0.20
17	Bd CX5461 10^-7M 4hr v Bd Control	-3.81	-8.44	0.82	-1.61	0.11	0.17

18	Bd CX5461 10^-7M 24hr v Bd Control	-14.00	-18.65	-9.35	-5.90	3.69E-09	1.85E-08
19	Bd CX5461 10^-7M 4hr v Bd CX5461 10^-7M 2hr	-0.28	-5.71	5.16	-0.10	0.92	0.95
20	Bd CX5461 10^-7M 24hr v Bd CX5461 10^-7M 2hr	-10.46	-15.52	-5.40	-4.05	5.04E-05	0.000151175
21	Bd CX5461 10^-7M 24hr v Bd CX5461 10^-7M 4hr	-10.19	-15.62	-4.75	-3.67	0.00024	0.00060
22	Bd CX5461 10^-6M 2hr v Bd Control	-10.21	-14.86	-5.55	-4.30	1.71E-05	5.70E-05
23	Bd CX5461 10^-6M 4hr v Bd Control	-11.74	-16.37	-7.11	-4.97	6.68E-07	2.86E-06
24	Bd CX5461 10^-6M 24hr v Bd Control	-22.50	-27.16	-17.85	-9.48	2.55E-21	1.53E-20
25	Bd CX5461 10^-6M 4hr v Bd CX5461 10^-6M 2hr	-1.53	-6.97	3.90	-0.55	0.58	0.68
26	Bd CX5461 10^-6M 24hr v Bd CX5461 10^-6M 2hr	-12.30	-17.36	-7.24	-4.76	1.90E-06	7.11E-06
27	Bd CX5461 10^-6M 24hr v Bd CX5461 10^-6M 4hr	-10.76	-16.20	-5.33	-3.88	0.00010	0.00028
28	Bd CX5461 10^-6M 2hr v Bd CX5461 10^-7M 2hr	-6.67	-11.73	-1.61	-2.58	0.0098	0.0172
29	Bd CX5461 10^-6M 4hr v Bd CX5461 10^-7M 4hr	-7.93	-12.99	-2.87	-3.07	0.0021	0.0040
30	Bd CX5461 10^-6M 24hr v Bd CX5461 10^-7M 24hr	-8.50	-13.56	-3.44	-3.29	0.00099	0.00211

G2 phase

ContrID	Contrast Name	Difference in percent of cells in G2 phase: Estimate	Difference in percent of cells in G2 phase: 95% lower confidence limit	Difference in percent of cells in G2 phase: 95% upper confidence limit	Z value	p-value	Benjamini-Hochberg adjusted p-value
1	Bp CX5461 10^-7M 2hr v Bp Control	2.38	-1.44	6.20	1.22	0.22	0.33
2	Bp CX5461 10^-7M 4hr v Bp Control	0.32	-3.47	4.11	0.17	0.87	0.90
3	Bp CX5461 10^-7M 24hr v Bp Control	8.12	4.30	11.94	4.16	3.13E-05	0.00010
4	Bp CX5461 10^-7M 4hr v Bp CX5461 10^-7M 2hr	-2.06	-6.75	2.63	-0.86	0.39	0.47
5	Bp CX5461 10^-7M 24hr v Bp CX5461 10^-7M 2hr	5.74	1.37	10.10	2.58	0.010	0.021
6	Bp CX5461 10^-7M 24hr v Bp CX5461 10^-7M 4hr	7.80	3.11	12.49	3.26	0.0011	0.0034
7	Bp CX5461 10^-6M 2hr v Bp Control	4.43	0.61	8.25	2.27	0.023	0.046
8	Bp CX5461 10^-6M 4hr v Bp Control	1.74	-2.05	5.53	0.90	0.37	0.46
9	Bp CX5461 10^-6M 24hr v Bp Control	41.17	37.34	44.99	21.11	6.19E-99	1.86E-97
10	Bp CX5461 10^-6M 4hr v Bp CX5461 10^-6M 2hr	-2.69	-7.38	2.00	-1.13	0.26	0.37
11	Bp CX5461 10^-6M 24hr v Bp CX5461 10^-6M 2hr	36.73	32.37	41.10	16.50	3.60E-61	5.03E-60

12	Bp CX5461 10^-6M 24hr v Bp CX5461 10^-6M 4hr	39.43	34.74	44.12	16.48	5.03E-61	5.03E-60
13	Bp CX5461 10^-6M 2hr v Bp CX5461 10^-7M 2hr	2.05	-2.31	6.41	0.92	0.36	0.46
14	Bp CX5461 10^-6M 4hr v Bp CX5461 10^-7M 4hr	1.42	-2.95	5.78	0.64	0.52	0.61
15	Bp CX5461 10^-6M 24hr v Bp CX5461 10^-7M 24hr	33.05	28.68	37.41	14.85	7.49E-50	5.62E-49
16	Bd CX5461 10^-7M 2hr v Bd Control	3.41	-0.61	7.42	1.66	0.096	0.15
17	Bd CX5461 10^-7M 4hr v Bd Control	4.00	0.01	7.99	1.96	0.050	0.083
18	Bd CX5461 10^-7M 24hr v Bd Control	4.30	0.29	8.31	2.10	0.036	0.063
19	Bd CX5461 10^-7M 4hr v Bd CX5461 10^-7M 2hr	0.59	-4.10	5.28	0.25	0.80	0.86
20	Bd CX5461 10^-7M 24hr v Bd CX5461 10^-7M 2hr	0.89	-3.47	5.26	0.40	0.69	0.76
21	Bd CX5461 10^-7M 24hr v Bd CX5461 10^-7M 4hr	0.30	-4.39	4.99	0.13	0.90	0.90
22	Bd CX5461 10^-6M 2hr v Bd Control	5.69	1.68	9.71	2.78	0.0054	0.013
23	Bd CX5461 10^-6M 4hr v Bd Control	10.78	6.79	14.77	5.29	1.21E-07	4.53E-07
24	Bd CX5461 10^-6M 24hr v Bd Control	18.07	14.06	22.08	8.83	1.08E-18	6.50E-18
25	Bd CX5461 10^-6M 4hr v Bd CX5461 10^-6M 2hr	5.09	0.40	9.78	2.13	0.033	0.063
26	Bd CX5461 10^-6M 24hr v Bd CX5461 10^-6M 2hr	12.38	8.01	16.74	5.56	2.70E-08	1.16E-07
27	Bd CX5461 10^-6M 24hr v Bd CX5461 10^-6M 4hr	7.29	2.60	11.98	3.05	0.0023	0.0058
28	Bd CX5461 10^-6M 2hr v Bd CX5461 10^-7M 2hr	2.29	-2.08	6.65	1.03	0.30	0.41
29	Bd CX5461 10^-6M 4hr v Bd CX5461 10^-7M 4hr	6.78	2.42	11.14	3.05	0.0023	0.0058
30	Bd CX5461 10^-6M 24hr v Bd CX5461 10^-7M 24hr	13.77	9.41	18.13	6.19	6.18E-10	3.09E-09

Bp: HCT116 BRCA2 proficient cells (Wild type strain)

Bd: HCT116 BRCA2 deficient cells (B18 BRCA2-/- strain)

Green color indicates statistically significant difference in pair-wise comparison.

Supplementary Table 7: Model estimates of fold change of amount of 45s pre-rRNA due to indicated drug for HCT116 wild type (WT) and BRCA2 knockout (*BRCA2*$^{-/-}$) cell genotypes.

Assay Type	Drug	Genotype omnibus Pr(>F)	Genotype omnibus Benjamini - Hochberg adjusted Pvalue	Genotype interaction Pr(>F)	Genotype main effect Pr(>F)	WT effect Abs FC Est	WT effect Direction	WT effect Abs 95% LCL	WT effect Abs 95% UCL	BRCA2-/- effect Abs FC Est	BRCA2-/- effect Direction	BRCA2-/- effect Abs 95% LCL	BRCA2-/- effect Abs 95% UCL
SYBRgreen	ACTINOMYCIN (50NM)	2.90E-42	5.22E-41	0.0117	2.77E-42	551.7118	Down	257.8001	1180.7055	137.2681	Down	64.1417	293.7643
Taqman	ACTINOMYCIN (50NM)	1.56E-55	1.88E-54	8.34E-06	1.04E-52	654.8909	Down	372.7603	1150.5573	100.2473	Down	56.8162	176.8778
SYBRgreen	BMH21 10^-6	1.09E-11	9.81E-11	0.4842	1.23E-12	19.6799	Down	10.0566	38.5119	19.6799	Down	10.0566	38.5119
SYBRgreen	BMH21 10^-7	0.0203	0.0522	0.1407	0.0174	1.6634	Down	1.0968	2.5225	1.6634	Down	1.0968	2.5225
Taqman	BMH21 10^-8	0.6226	0.8301	0.3683	0.7156	1.1277	Down	0.5850	2.1736	1.1277	Down	0.5850	2.1736
SYBRgreen	CX3543 10^-5	7.47E-08	3.36E-07	0.1128	3.27E-08	12.1411	Down	5.4989	26.8065	12.1411	Down	5.4989	26.8065
Taqman	CX3543 10^-5	6.72E-07	2.69E-06	0.0684	4.94E-07	11.8462	Down	4.9064	28.6022	11.8462	Down	4.9064	28.6022
SYBRgreen	CX3543 10^-6	0.0004	0.0015	0.0710	0.0004	2.1086	Down	1.4130	3.1467	2.1086	Down	1.4130	3.1467
SYBRgreen	CX3543 10^-7	0.2171	0.3908	0.8031	0.0824	1.3627	Down	0.9599	1.9345	1.3627	Down	0.9599	1.9345
Taqman	CX3543 10^-8	0.3761	0.5641	0.4338	0.2454	1.4633	Down	0.7646	2.8004	1.4633	Down	0.7646	2.8004
SYBRgreen	CX5461 10^-5	2.40E-11	1.44E-10	0.0005	1.20E-09	28.0726	Down	12.3538	63.7920	3.3109	Down	1.4570	7.5237
Taqman	CX5461 10^-5	8.60E-09	5.16E-08	0.0011	2.47E-07	31.9583	Down	11.6204	87.8919	2.7546	Down	1.0016	7.5757
SYBRgreen	CX5461 10^-6	0.1172	0.2345	0.5499	0.0466	1.5111	Down	1.0064	2.2691	1.5111	Down	1.0064	2.2691
SYBRgreen	CX5461 10^-7	0.8038	0.8038	0.5167	0.9093	1.0211	Up	0.7094	1.4697	1.0211	Up	0.7094	1.4697
Taqman	CX5461 10^-8	0.7660	0.9192	0.6621	0.5579	1.2298	Down	0.6096	2.4809	1.2298	Down	0.6096	2.4809

Key:	
(light green)	Results showing statistically significant absolute fold change (greater than 1.0) in either BRCA2 proficient or deficient genotypes, after multiple comparisons adjustment.
(dark green)	Different assay types targeting same biomarker.
(white)	Results failing to show statistically significant absolute fold change.

Supplementary Table 8. ChIP-seq library reads count, alignment and filtering statistics

Sample ID	treatment	dosage	target	reads	mapped	map_rate	hqread	hq_rate
1	CX4561	0	IgG	2228050	1896071	0.851	1387694	0.7319
2	CX4561	0	IgG	2883594	2310336	0.8012	1595316	0.6905
3	CX4561	10^-7	IgG	2498292	1238154	0.4956	846882	0.684
4	CX4561	10^-7	IgG	3035486	1658590	0.5464	1169154	0.7049
5	CX4561	10^-7	IgG	2455396	1585449	0.6457	1178560	0.7434
6	CX4561	0	Rad51	2114440	1517745	0.7178	1035516	0.6823
7	CX4561	0	Rad51	2050588	1207796	0.589	722154	0.5979
8	CX4561	10^-7	Rad51	2823816	849686	0.3009	496966	0.5849
9	CX4561	10^-7	Rad51	2324324	703805	0.3028	392782	0.5581
10	CX4561	10^-7	Rad51	3163588	1644117	0.5197	1209006	0.7354

10 libraries with 4 conditions were created, some in biological duplicates, others in triplicates. Each library (sample ID) was treated with CX-5461 (treatment) at either 10^{-7}M or vehicle, after which a pull down was performed with the control non-specific IgG or anti-Rad51 antibody (target). For each library, we report the number of FASTQ reads produced (reads), successfully aligned to hg19 by bowtie2 (mapped), and the alignment rate (map_rate). Finally, we keep only concordantly aligned paired reads with a mapping quality >= 30, reporting the number of reads (hqread) and the percentage of aligned reads that were kept (hq_rate).

Supplementary Table 9: MACS2 peak statistics on RAD51-ChIP samples.

Signal	Back ground	Signal target	Signal dosage	Background target	Background dosage	peaks	Replicate peaks	Replicate Peaks percentage
3	1	IgG	10^-7	IgG	0	4216	551	0.13
4	1	IgG	10^-7	IgG	0	5960	586	0.09
5	1	IgG	10^-7	IgG	0	2790	258	0.09
6	1	Rad51	0	IgG	0	3289	109	0.03
7	1	Rad51	0	IgG	0	2885	131	0.04
8	1	Rad51	10^-7	IgG	0	14406	6134	0.42
9	1	Rad51	10^-7	IgG	0	11323	5411	0.48
10	1	Rad51	10^-7	IgG	0	4505	2463	0.55
3	2	IgG	10^-7	IgG	0	1815	98	0.05
4	2	IgG	10^-7	IgG	0	2459	73	0.03
7	2	Rad51	0	IgG	0	1030	32	0.03
8	2	Rad51	10^-7	IgG	0	7577	2348	0.31
9	2	Rad51	10^-7	IgG	0	5676	2071	0.36
10	2	Rad51	10^-7	IgG	0	2410	935	0.39

Using the duplicate untreated IgG library as a background, peaks were called with MACS2 for the treated Rad51 and IgG libraries, along with the untreated Rad51 libraries. Each pairing is uniquely identified by the sample ID (Supplementary Table 8), denoted in the signal and background columns, and detailed in signal target, signal dosage, background target, and background dosage. The total number of peaks with an FDR value >= 0.1 are reported (peaks), and any peak that occurs in at least one other replicate library (within +/-500 bp of peak start and end), is consider a replicate peak (replicate peaks). We report the percentage of replicate peaks per sample in replicate peaks percentage.
